# The second law of thermodynamics as variation on a theme of Carathéodory

**DOI:** 10.1098/rspa.2021.0425

**Published:** 2021-09

**Authors:** Joe D. Goddard

**Affiliations:** Department of Mechanical and Aerospace Engineering, University of California, San Diego, 9500 Gilman Drive, La Jolla, CA 92093-0411, USA

**Keywords:** second law of thermodynamics, entropy and temperature, third law, thermostatic stability, convexity, non-equilibrium entropy, Clausius–Duhem inequality

## Abstract

This paper revisits the second law of thermodynamics via certain modifications of the axiomatic foundation provided by the celebrated 1909 work of Carathéodory. It is shown that his postulate of *adiabatic inaccessibility* represents one of several constraints on the energy balance that serve to establish the existence of thermostatic entropy as a foliation of state space, with temperature representing a force of constraint. To achieve the thermostatic version of the second law, as embodied in the postulates of Clausius and Gibbs, work principles are proposed to define thermostatic equilibrium and stability in terms of the convexity properties of internal energy, entropy and related thermostatic potentials. Comparisons are made with the classic work of Coleman and Noll on thermostatic equilibrium in simple continua, resulting in a few unresolved differences. Perhaps the most novel aspect of the current work is an extension to irreversible processes by means of a non-equilibrium entropy derived from recoverable work, which generalizes similar ideas in continuum viscoelasticity. This definition of entropy calls for certain revisions of modern theories of continuum thermomechanics by Coleman, Noll and others that are based on a generally inaccessible entropy and undefined temperature.

## 1. Introduction

The landmark paper of Carathéodory [1] ranks among the more renowned in the field of thermodynamics.^1^ Assessments of his work range from generallylaudatory [2–10] to occasionally dismissive [11–14], with the article [15] raising questions as to priority.

The highlight of Carathéodory’s treatment is the derivation of thermostatic entropy and temperature from the postulate of *adiabatic inaccessibility*. This is to be contrasted with more recent treatments, devoted primarily to continuum thermomechanics, by Day and co-workers [16–18]. In particular, they base the existence of entropy on considerations of work and thermal efficiency, which implicitly involve elements of the Kelvin–Planck arguments. Beyond these macroscopic or continuum-level models are the numerous ‘microscopic’ theories that define thermostatic and non-equilibrium entropy in terms of *internal variables*, e.g. [19–21]. Maugin ([22], Ch. 5, Section 5.3) provides an admirable summary (preceded by a provocative assessment of *rational thermodynamics*). In the present article, we set aside such models as well as various forms of near-equilibrium thermodynamics (e.g. the ‘TIP’ also discussed by Maugin).

For thermostatic entropy, the present article favours the path taken by Carathéodory, reflecting the view of Uffink [9, pp. 308–309] that ‘despite a number of original defects, the approach pioneered by Carathéodory has in recent years turned out to be the most promising route to obtain a clear formulation of the second law’. The words ‘route to’ are crucial, reflecting the opinion of several authors, present company included, that Carathéodory’s proof of the existence of entropy and temperature, does not *per se* achieve a complete formulation of the second law, in particular by a failure to provide further restrictions on entropy based on clearly defined fundamental principles. This deficiency is minimized by Lieb & Yngvason [23], who accept the existence of entropy as tantamount to the second law, while conceding that this is at odds with conventional views.

As to the second law, Uffink provides an entertaining survey [9], recalling PW Bridgman’s lament that there are almost as many formulations as there are discussions of it. Examples of the latter include the survey by Pogliani & Berberan-Santos [3], who review the history of various reactions to Cararathéodory’s work, and Honig [24], who offers a physico-chemical perspective and who, like others before him [25], weighs its merits relative to the Kelvin–Planck version. Sneddon [26] offers an applied mathematical perspective on the integrability of Pfaffian forms pursued by Carathódory. The volume edited by Kestin [27] offers a collection of key historical papers, with English translations of some, and Delphenich [28] provides English translations of others. More recent edited volumes [20,29] offer a broad survey of entropy-related topics, ranging from statistical mechanics to the continuum thermodynamics of non-equilibrium systems, much of which is only tangentially relevant to the present treatment.

Closest in spirit if not in method to the present work is the mathematical treatment of the second law presented in Frankel’s treatise [8], whose title suggests the apt description of Carathéory’s work as *geometric thermodynamics* (cf. Maugin [10]), which in effect provides an axiomatic foundation for the thermodynamic surfaces of Gibbs. (Rendered palpable by Maxwell, these are illustrated in the encyclopedia article [30], which, incidentally, contains a wealth of interesting historical references too numerous to cite in the present paper.)

In a chapter entitled ‘Holonomic and non-holonomic constraints’, Frankel [8, Ch. 6] builds on Carathéodory’s work by identifying entropy as a global *adiabatic foliation* of state space, guaranteed by the holonomy of entropy according to a proof cited elsewhere [31, p. 376]. However, Frankel mainly focuses on the thermostatics of fluids with restriction to p-v work, and his reliance on Kelvin’s treatment (which he dubs ‘Kelvin implies Carathéodry’) runs counter to the approach of the present work, which is based on the premise that the Kelvin–Planck treatment should follow from a properly formulated version of the second law for the simple systems envisaged by Carathéodory.

As is the case with many writers going back to Clausius and Gibbs, Frankel appeals to irreversible processes (e.g. stirring) in his treatment of thermostatics of fluids. Aside from these minor criticisms, Frankel’s focus on constraints is fairly novel and also germane to the present work, in which we re-state Carathéodory’s treatment in terms of holonomic constraints.

Given the observation of Bridgman cited above, the reader may well ask why yet another formulation of the second law. As partial answer, I note that the goals are manifold. First, as a sort of update of the existing literature, I provide a review and synthesis of ideas found in key articles dealing with Carathéodory’s theory. This review is selective and by no means exhaustive but I hope will serve as attraction and guide to readers who have more than a passing interest in the subject. Second, I propose a pair of *thermomaterial* work principles, in order to justify the conditions of equilibrium and stability laid down by Gibbs, based solely on properties of convexity for energy, entropy and other thermodynamic potentials, and I compare these with the treatment of continuum thermostatics by Coleman & Noll [32].

The culmination of the present paper is based on a long-standing malaise, shared notably by Meixner [33,34] (cf. Maugin [22], Section 5.3), with the formulation of the second law based on the *Clausius–Duhem inequality* in the form employed almost universally in the contemporary thermomechanics of continua. In particular, this formulation appears to rely on concepts of entropy and temperature borrowed from equilibrium thermodynamics, i.e. thermostatics, in various treatments of irreversible processes. (Conversely, some of the classical literature involves an invocation of irreversibility to extend the reach of thermostatics.) To address this state of affairs, I shall offer a definition of a history-dependent non-equilibrium entropy that replaces a generally undefined non-equilibrium temperature with the measurable property of internal energy. This provides a connection to certain contemporary efforts to derive Helmholtz free energy and, hence, entropy from continuum viscoelasticity [35–37].

The present article does not rely on a progression through formal mathematical proofs but rather recalls a few existing proofs insofar as they relate to some requisite mathematical concepts and techniques. The intent is to offer a logical albeit not always mathematically rigorous treatment of the second law, which the author hopes may appeal to a diverse readership, while perhaps motivating a more rigorous treatment by those who are so inclined.

## 2. Recapitulation, with variations, of Carathéodory’s work

We recall that Carathéodory [1] considers heterogeneous systems of several communicating phases and imposes nominal ‘equilibrium’ conditions to reduce the number of variables. Without adopting his *ab initio* imposition of such conditions, we shall adopt his definition of a *simple* system as one whose static thermodynamic state is specified by a finite set of n+1 independent variables that he denotes by $x_{0},x_{1},\ldots ,x_{n}$, of which the last n serve to define generalized work. In the following, we employ the term *thermomaterial* as the extension of *thermomechanical* to this and other forms of work (electrostatic, magnetostatic, chemical, etc.). Subject to later variants, we adopt the alternative notation $z^{0},z^{1},\ldots ,z^{n}$ for the independent state variables, with superscripts representing the usual notation for coordinates or components of tangent vectors to a manifold, and with subscripts labelling generalized forces as duals.

While Carathéodory’s designation of thermostatic states as ‘equilibrium’ is consistent with the conventional term ‘equilibrium thermodynamics’, it seems logically out of place prior to a definition of entropy and associated extremum principles of the type postulated by Clausius [38–40] and Gibbs [27,41]. Furthermore, as done by many subsequent authors, including [8, Ch. 6 and 15], Carathéodory refers to equations of state that are in fact derivable from functions involving entropy, prior to the derivation of such functions from principles of equilibrium. (For example, the p-V-T equations of state for fluids and the stress–strain relations for elastic solids are derivable from Helmholtz free energy.)

As assumed by others, e.g. [8, Ch. 6] and [15], Carathéodory assumes quasi-static processes to be reversible, an assumption invalidated by various rate-independent ‘frictional’ processes, exemplified, e.g. by the continuum theory of plasticity, and recognized long ago by Gibbs ([42, pp. 58–59] and [43, pp. 147–148])^2^ and Bridgman [44], followed by subsequent authors (cf. Maugin [22], Section 53), and the same is true of rate-independent magnetostatic dissipation and hysteresis. The restriction to quasi-static processes also ignores the possibility of an instantaneous elastic response that satisfies thermostatic relations according to Coleman [45] (which in principle would allow one to define a non-equilibrium entropy for arbitrary histories of deformation for a broad class of materials, *provided temperature is taken as a primitive variable*).

Here, as in the following, we employ the term *thermostatic* to denote the conventional *thermodynamic-equilibrium* states, to be distinguished from quasi-static processes, which, as pointed out above, need not be the reversible processes to be defined below.

### (a) Constraints on the first law

The above considerations motivate a more concrete treatment in which we take $z^{0}=\varepsilon$, following Meixner [46] and Frankel [8, p. 179], where ε denotes specific internal energy. As is the case with the remaining configurational variables, ε>0 is assumed to be a measurable quantity, i.e. an *observable*, at least relative to some well-defined reference state, or else connected to measurable quantities via the first law, as emphasized by Tisza [47]. Tisza goes a step further and demands that all the configurational variables represented here by x are quantities conserved during exchanges or interactions between different systems, whereas we shall allow for more general coupling between configurational variables, with energy conserved via reversible holonomic couplings. Thus, our interacting systems may possess different configurational variables, as is the case in the interaction of solids with fluids. As a weaker requirement, we assume internal energy is additive over subsystems and that the set of configurational variables is the union of configurational variables for subsystems, with additivity defining *extensive variables*. Once a definition of equilibrium is established, we assume it will suffice to establish entropy as additive over subsystems, which is treated as axiomatic by others.

Next, we write

2.1 $$\text{z}=\{z^{0},z^{1},\ldots ,z^{n}\}=\{\varepsilon ,\text{x}\},\;\text{with}\;z^{0}=\varepsilon ,\;\text{and}\;\text{x}=\{x^{1},x^{2},\ldots ,x^{n}\}.$$

This serves to define the thermodynamic state space T as an n+1-dimensional manifold that we may take to be homeomorphic or, as assumed by Carathéodory [1], identical with $R^{n+1}$. Then the incremental heat đ*q* received by the system on any infinitesimal trajectory is given by the Pfaffian differential form, i.e. the one-form, involving conjugate forces $f_{i}(\text{z}),\;i=1:n$ and representing the first law (or energy balance, taken as axiomatic):

2.2 $$đq=\text{d}\varepsilon +đw=\text{q}\cdot \text{d}\text{z}=\sum_{i=0}^{n}q_{i}(\text{z})\;\text{d}z^{i},\;\text{where}\;đw=\text{f }\cdot \text{d}\text{x}=\sum_{i=1}^{n}f_{i}(\text{z})\;\text{d}x^{i},\;\text{and}\;\text{q}=\{1,\text{f}\},$$

where we adopt the conventional *active* definition of incremental work đw as work done by the system. Those who prefer may simply omit all the symbols đ shunned by the strict adherents of exterior calculus, e.g. [8, p. 180].

As signalled by (2.2), we shall in the following adopt with slight variation the notational conventions of Gibbs [41,42] passed down to the modern school of *rational thermodynamics*, whereby lower case Greek letters denote various thermodynamic properties that can be regarded as total or specific (per unit mass) values in the case of extensive variables.

A scalar constraint on the first law is then defined by the one-form

2.3 $$đ\sigma =\sum_{i=0}^{n}\sigma_{i}(\text{z})\;\text{d}z^{i}=0,$$

so that (2.2) can be rewritten as

2.4 $$đq=\sum_{i=0}^{n}[q_{i}(\text{z})-\lambda \sigma_{i}(\text{z})]\;\text{d}z^{i},$$

where λ is a Lagrange multiplier. As an aside, we note that Frankel ([8], Sect. 6.2b) treats a vector of constraints, the ‘Mayer-Lie system’, involving a vector of Lagrange multipliers. Following Carathéodory and the treatment below, these might be construed to represent constraints on subsystems of a given system (which might involve couplings between state variables), or they could represent multiple constraints on processes defined by the first law, a matter consider briefly at the end of this section.

In the case of a single, smooth *holonomic* constraint, specified by a manifold σ(z)= const., we may take $\sigma_{i}=\partial_{i}\sigma (\text{z}),\;i=0:n$, where $\partial_{i}=\partial /\partial z^{i}$. As a patently obvious example, the constraint $\sigma =x^{k}$= const. yields $\lambda =f_{k}$ as reaction against the constraint, with đw and đ*q* restricted accordingly. As a special case mentioned below is the *isenergic* process, $z^{0}=\varepsilon =\text{const.}$, with $\lambda =q_{0}=1$ and đq=đw.

#### (i) Adiabaticity

With all due respects to Carathéodory’s physically descriptive term *adiabatic inaccessibility*, we simply assert here that the *constraint of adiabaticity*, đq=0, *is holonomic*, where đ*q* is the one-form given in (2.2). This represents the restriction to an n-dimensional submanifold S of T representing a family (or foliation [8,31]) of hypersurfaces η(z)= const. It is then evident from (2.4) that $\lambda^{-1}$ represents an integrating factor for the differential form đ*q* in (2.2), with $\partial_{i}\eta =f_{i}/\lambda$.

To amplify on the above, it is worth repeating the classical condition of integrability,

2.5 $$\partial_{i}\partial_{j}\eta =\partial_{i}(q_{j}/\lambda )=\partial_{j}(q_{i}/\lambda )=\partial_{j}\partial_{i}\eta ,\;\text{for}\;i,j=0:n,$$

which, by means of a bit of elementary calculus, can be rearranged to read

2.6 $$(q_{j}\partial_{i}-q_{i}\partial_{j})\chi =\partial_{i}q_{j}-\partial_{j}q_{i},\;\text{where}\;\chi =\log\lambda .$$

To facilitate subsequent algebraic manipulations, rewrite this as

2.7 $$\Omega_{ij}:=(\nabla_{i}-\nabla_{j})\chi =\nabla_{i}\chi_{j}-\nabla_{j}\chi_{i},\;\text{where}\;\chi_{k}=\log q_{k},\;\text{and}\;\nabla_{k}=\frac{1}{q_{k}}\partial_{k},\;k=0:n.$$

Although not necessary, we can provisionally regard $\nabla_{k}$ as components of a gradient operator on an orthogonal curvilinear system $z^{0},\ldots z^{n}$ with $q_{k}$ serving as metrical coefficients (with ${\left\{\sum_{i=0}^{n}q_{i}^{2}{\left(\text{d}z^{i}\right)}^{2}\right\}}^{1/2}$ representing an energy norm). It follows that

2.8 $$(\nabla_{i}\chi_{j}-\nabla_{j}\chi_{i})+(\nabla_{j}\chi_{k}-\nabla_{k}\chi_{j})+(\nabla_{k}\chi_{i}-\nabla_{i}\chi_{k})=\Omega_{ij}+\Omega_{jk}+\Omega_{ki}=0.$$

Hence, reverting to the original symbols $q_{i},\partial_{i}$ we obtain the relations:

2.9 $$q_{i}(\partial_{j}q_{k}-\partial_{k}q_{j})+q_{j}(\partial_{k}q_{i}-\partial_{i}q_{k})+q_{k}(\partial_{i}q_{j}-\partial_{j}q_{i})=0,$$

given e.g. by Frankel ([8, p. 174], with r=1) and Honig ([24], eq. (9.2.20)). These can be expressed as q∧dq=0 in the language of exterior calculus employed by Carathéodory [1,8], who identifies η(z) as entropy and λ=θ(z) as temperature, with đq=θdη. Thus, with η interpreted as a new configurational variable, θ represents the reactive force of constraint to the hypersurfaces of constant η.

Taking θ to be the empirical absolute temperature of thermodynamics, with (as axiom) θ>0, ensures the positivity of ${\left(\partial \eta /\partial \varepsilon \right)}_{\text{x}}=1/\theta$ and the invertibility of η(ε,x), with inverse function $\hat{\varepsilon }(\eta ,\text{x})$. Following the contemporary literature [32,48], we refer to the latter as the *caloric equation of state*. Then, the energy balance or first law of thermodynamics reduces to the general Gibbs [42] relation

2.10 $$\text{d}\hat{\varepsilon }=\hat{\text{f}}\cdot \text{d}\hat{z}=\theta \text{d}\eta -\text{f}\cdot \text{d}\text{x},\;\text{with}\;\theta =\partial_{\eta }\varepsilon ,\;\text{f}=-\partial_{\text{x}}\varepsilon ,\;\hat{z}=\{\eta ,\text{x}\},\;\hat{\text{f}}=\{\theta ,-\text{f}\}=\partial_{\hat{z}}\hat{\varepsilon }.$$

In (2.10), we have introduced a generalized force {θ,−f} conjugate to the displacement {dη,dx}, i.e. producing a generalized *thermomaterial work*. This extended notion of work, which we exploit in the stability analysis to follow, is based on a view of θdη as *inner working* (consistent with Clausius’s notion and nomenclature ‘entropy’ for internal change [49]), as opposed to the *extrinsic* or *material* variety defined by $f_{i}\;\text{d}x^{i}$. In that respect, it may be viewed as the analogue of the mechanical work of isotropic expansion pdv, with pv/R equivalent to θ and Rdln⁡v equivalent to dη, where v is the continuum-level manifestation of Boltzmann’s famous statistical-thermodynamic measure ‘W’ that can be interpreted in terms of a volume in microscopic phase space.

As a variant on the treatment of Coleman & Noll [32] we shall adopt the caloric equation of state and (2.10) as the first criterion for *thermostatic equilibrium*, with the second criterion to be discussed below. Furthermore, we define a *reversible process* as one governed by (2.10).

#### (ii) Alternative constraints

Here, we consider an alternative to Carathéodory’s adiabatic constraint, which in effect yields no new results but rather shows that many roads lead to Rome.^3^ Hence, consider the constraint

2.11 $$đw=\sum_{i=1}^{n}f_{i}(\varepsilon ,\text{x})\;\text{d}x^{i}=0,$$

which for want of a better term might be dubbed *potentiostatic*, as it involves *potentiostatic inaccessability* of states near a given state. While not holonomic, it implies provisionally the existence of a state function σ(ε,x) such that $\lambda f_{i}={\left(\partial_{i}\sigma \right)}_{\varepsilon },\;i=1:n,$, with integrating factor λ. As in the following sections, we employ the notation ${\left(\;\;\right)}_{c}$ to denote changes with quantity c(z) held constant.

Then, with or without the constraint (2.11), the energy balance reads

2.12 $$\begin{array}{rl} \text{d}\varepsilon & =đq-\lambda^{-1}\sum_{i=1}^{n}{\left(\partial_{i}\sigma \right)}_{\varepsilon }\text{d}x^{i}=đq-\lambda^{-1}{\left(\text{d}\sigma \right)}_{\varepsilon }\\ & =đq-\lambda^{-1}(\text{d}\sigma -\partial_{\varepsilon }\sigma \text{d}\varepsilon ),\;\text{or}\;đq=\text{d}\varepsilon +\lambda {\left(\text{d}\sigma \right)}_{\varepsilon }, \end{array}$$

which is satisfied by taking $\lambda =\partial_{\varepsilon }\sigma$ and $đq=\lambda^{-1}\text{d}\sigma$. We can then identify σ with the entropy η and $\lambda^{-1}$ with θ.

We note that as another indirect path to entropy, one may consider the obviously holonomic constraint dε=0, which implies that đq−đw=0 reflects a holonomic constraint, with $đq=\lambda \partial_{\varepsilon }\sigma$ and $f_{i}=-\lambda \partial_{i}\sigma$. Although we shall not pursue the matter here, it is plausible that the above treatment of constraints could be effected by an appeal to simultaneous constraints on a pair chosen from {q,ε,w}, with restriction to an (n−1)-dimensional manifold. Then, according to the Frobenius theorem, and in the language of differential forms, integrability requires that a selected pair of one-forms $\left\{\omega_{1},\omega_{2}\right\}$ satisfy $\text{d}\omega_{i}\wedge (\omega_{1}\wedge \omega_{2})=0$, for i=1,2, [8, p. 168] or [49, p. 114].

### (b) A construct for entropy

Given the constraint (2.11), we can write

2.13 $${\left(\text{d}\eta \right)}_{\text{x}}=\theta^{-1}\text{d}\varepsilon \;\text{and}\;∴\eta (\varepsilon ,\text{x})=\int_{\varepsilon_{0}}^{\varepsilon }\theta {\left(\varepsilon^{\prime },\text{x}\right)}^{-1}\text{d}\varepsilon^{\prime }+\eta_{0}(\text{x}),$$

where the integral is taken at fixed x. Complete integrability requires that $\partial_{\text{x}}\partial_{\varepsilon }\eta =\partial_{\varepsilon }\partial_{\text{x}}\eta$ (a Maxwell relation), so that we must take $\eta_{0}$ to be a constant independent of x. This can be considered as an new restriction that leads eventually to the third law of thermodynamics.

The integral (2.13), given in effect by Clausius [39] for fluids, provides a primitive construct for entropy derived from calorimetric data, such as sensible heat defined by specific heat $c_{\text{x}}$ and by latent heats representing discontinuities in ε at phase transitions, where the integral is be interpreted as a Riemann–Stieltjes integral yielding the corresponding discontinuities in η. The interpretation of $\varepsilon_{0}$ and $\eta_{0}$ are subject to various forms of the third law of thermodynamics.

Since ordinary calorimetry involves a measureable and presumably equilibrium temperature θ, it is necessary to introduce an equation of state $\varepsilon =\overset{ˇ}{\varepsilon }(\theta ,\text{x})$, the inverse of θ(ε,x), with

2.14 $$\eta (\theta ,\text{x})=\int_{\theta_{0}}^{\theta }c_{\text{x}}(\theta^{\prime },\text{x})\frac{\text{d}\theta^{\prime }}{\theta^{\prime }}+\eta_{0},\;\text{where}\;c_{\text{x}}(\theta ,\text{x})=\partial_{\theta }\overset{ˇ}{\varepsilon },$$

and with, once again, appropriate modification for discontinuities at phase boundaries. (These can be formally treated by representing $c_{\text{x}}$ as Dirac delta in the normal direction to a phase boundary or as a weaker singularity at thermodynamic critical points.) We recall that the classical third law of thermodynamics sets $\eta_{0}=0$ at $\theta_{0}=0$ for perfectly crystalline solids.

Having defined entropy and temperature along the lines laid down by Carathéodory, we turn to the question of equilibrium and stability. We make a distinction in the following between equilibrium and stable equilibrium, in contrast to Coleman& Noll’s [32] treatment of the thermomechanics of simple continua. From the latter perspective, the simple system of Carathéodory should be regarded as local, applying to a homogeneous piece of a continuum subject to a homogeneous deformation or, equivalently, to a material point in a simple continuum, to be contrasted with an inhomogeneous body subject to various boundary conditions. Coleman& Noll [32] reserve the term ‘stability’ for the latter situation, but this unfortunately rules out a consideration of local *material instability* of homogeneous states arising from the loss of convexity (defined below), which we recall leads to Ball’s ‘singular minimizers’ [50] and phase transitions. In addition to its locality, the stability to be considered here is generally infinitesimal rather than finite although extensions to finite stability are evident.

## 3. Equilibrium, extremality and stability

As indicated above, the term ‘equilibrium’ is employed by Carathéodory, not to mention numerous other investigators, prior to actually establishing precise criteria for equilibrium, such as those postulated by Gibbs [41] and derived next from a general work principle.

### (a) Equilibrium via constraints

First, note that the thermostatic energy balance (2.10) can be cast into a variational form

3.1 δε−θδη=−δw:=−f⋅δx,

which may be regarded as one constraint on equilibrium processes. As standard notation δ indicates an infinitesimal change arising from variations of a set of n+1 independent variables subject to any number <n+1 of constraint. Then, as an extension of the classical condition of mechanical equilibrium, as e.g. formulated by Moreau ([51], Eqn (3.2)) in terms of virtual velocities, we assert that a system is at local thermostatic equilibrium provided that (3.1) is satisfied, subject to the inequality constraint δw=f⋅δx≤0 and any additional constraints that may be imposed on a process. The former stipulates that no work can be obtained from the system subject to the remaining constraints and, in the absence of the latter, the relation (3.1) leads immediately for θ>0 to the extrema postulated by Gibbs [41,42], which in his notation read:

3.2 $${\left(\delta \eta \right)}_{\varepsilon }\leq 0,\;{\left(\delta \varepsilon \right)}_{\eta }\geq 0,\;\text{and}\;{\left(\delta \psi \right)}_{\theta }\geq 0,\;\text{where}\;\psi =\varepsilon -\theta \eta ,$$

with ψ=ψ(θ,x) denoting Helmholtz free energy. For the applications to follow, note that the energy balance yields the well-known result $\text{f}=-{\left(\partial_{\text{x}}\psi \right)}_{\theta }$ that identifies ψ as the isothermal work potential. The work-principle enunciated above seems to provide a foundation for the theorems enunciated by Gibbs [42, p. 56].

With δ replaced by Δ to denote a finite change of state, Gibbs [42, p. 57] proposes the inequalities (3.2) as criteria for stable equilibrium. In that respect, it is worth recalling that, in the present notation, the criterion for local thermostatic equilibrium of a simple continuum proposed by Coleman & Noll ([32], Eq. (7.3)) reads

3.3 $$\Delta \varepsilon +\text{f}\cdot \Delta \text{x}-\theta \Delta \eta >0,\;\text{with, therefore, }\;{\left(\Delta \varepsilon \right)}_{\eta ,\text{x}}>0,\;{\left(\Delta \eta \right)}_{\varepsilon ,\text{x}}<0,\;\&\;{\left(\Delta \psi \right)}_{\theta ,\text{x}}>0,$$

which corresponds to the condition of stable equilibrium of Gibbs.

If one replaces the strict inequality > in (3.3) by ≥ the relation remains valid in the infinitesimal limit Δ→δ where the inequality gives way to equality and reduces to (3.1), which, e.g. represents the equation of Gibbs ([42], pp. 184 ff., eqn (355)) for the thermostatic equilibrium of a solid.

As Coleman and Noll no doubt appreciate, (3.3) dictates the strict convexity of the caloric equation of state ε=ε(η,x), which when differentiable gives:

3.4 $$\Delta \varepsilon +\text{f}\cdot \Delta \text{x}-\theta \Delta \eta =\Delta \varepsilon -\Delta \text{x}\cdot \partial_{\text{x}}\varepsilon -\Delta \eta \partial_{\eta }\varepsilon =\Delta \varepsilon -\Delta \text{z}\cdot \partial_{\text{z}}\varepsilon >0,$$

where $\text{z}=\hat{\text{z}}$, as defined in (2.10), and $\partial_{\text{z}}\varepsilon$ is evaluated at the initial point ${\text{z}}_{1}$ of a path, with $\Delta \text{z}={\text{z}}_{2}-{\text{z}}_{1}$. Positivity of the last two expressions in (3.4) represents the standard condition for convexity. The endpoints may be interchanged leading to the relation analogous to that given by Hill [52]:

3.5 $$(\Delta \partial_{\text{z}}\varepsilon )\cdot \Delta \text{z}=\Delta \hat{\text{f}}\cdot \Delta \text{z}>0,$$

which is a finite version of the infinitesimal work-principle proposed below.

In order to secure agreement of (3.3) with (3.1) and the analysis presented below, one must replace the strict inequality in (3.3) by ≥ and add the constraint of constant x to the Gibbs criteria (3.2), which otherwise might be interpreted as valid for the extended homogeneous phases of matter envisaged by Gibbs. Alternatively, one may impose the inequality constraint f⋅δx≤0 proposed above in order to obtain (3.2) from (3.3).

We recall that Coleman & Noll ([32], eqn (9.2)) propose as an alternative criterion for equilibrium the strict concavity of entropy, i.e. of the function η(ε,x), the original form for entropy as state function whose inverse is the caloric equation of state. We consider next the stability of equilibrium, with no further attempt to reconcile the different definitions of equilibrium, given that the literature on nonlinear elasticity abounds with proposed restrictions on stress–strain relations [53] (mainly aimed at securing uniqueness of solution to elastostatic boundary-value problems).

As one additional point, note that the analysis of Coleman & Noll [32] applies to simple continua, in which the configuration variable x denotes the local deformation gradient and the conjugate force −f the Piola–Kirchhoff stress. By contrast, the present treatment encompasses possible extensions to structured and polar continua, by enlarging the set of configurational variables and conjugate forces to include higher deformation gradients, Cosserat effects and conjugate hyperstresses. The same is true of systems with varying chemical composition, mentioned but not addressed by [32], where the relevant forces are the chemical potentials of Gibbs. Such modifications would seem more or less obvious according to the present formulation.

### (b) Infinitesimal convexity and stability

A brief summary of well-known Legendre–Massieu forms and convexity relations is relegated to the appendix, as we have limited need of these in the following discussion.

With equilibrium defined by the condition (3.1), we define stability of equilibrium in terms of *generalized thermomaterial work* with (2.10) cast in the variational form:

3.6 $$\delta \varepsilon (\text{z})=\hat{\text{f}}\cdot \delta \text{z}=(\partial_{\text{z}}\varepsilon )\cdot \delta \text{z},$$

where for simplicity we drop the circumflexes on $\left\{\hat{\varepsilon },\hat{z}\right\}$ here and in most of the following. Then, the second variation of ε given by a *second-order thermomaterial work* provides the analogue of the classical (Legendre–Hadamard [54]) mechanical stability criterion:

3.7 $$\delta^{2}\varepsilon =\delta \hat{\text{f}}\cdot \delta \text{z}=\delta \text{z}\cdot (\partial_{\text{z}}\partial_{\text{z}}\varepsilon )\cdot \delta \text{z}=\delta \text{z}\cdot \text{H}\cdot \delta \text{z}\geq 0,\;\text{with}\;\text{H}=\partial_{\text{z}}⊗\partial_{\text{z}}\varepsilon =\{\partial_{\text{x}},\partial_{\eta }\}⊗\{\partial_{\text{x}},\partial_{\eta }\}\varepsilon ,$$

where ⊗ denotes the tensor product and where the resulting matrix H is the *Hessian* of ε(x,η).

The expression following the first equal sign in (3.7), obviously an infinitesimal version of (3.5), represents a generalization of the classical thermomechanical stability criterion requiring H to be non-negative definite whenever ε possesses the partial derivatives involved; cf. the seminal article by Hill [52] and the more recent summary by Lubarda [55] on thermoelasticity.

Given the convexity of ε(η,x) and the fact that it is a strictly increasing function of η, because $\partial_{\eta }\varepsilon =\theta >0$, it follows that the inverse function η(ε,x) is concave with respect to ε [56]. The concavity of η with respect to x follows from our equilibrium criterion f⋅δx≤0, since

3.8 $${\left(\partial_{i}\varepsilon \right)}_{\eta ,x^{j\neq i}}\delta x^{i}=-f_{i}\delta x^{i}\geq 0,\;(\text{no sum on}\;i),$$

so that ε is a non-decreasing function of each of the configurational coordinates $x^{i}$. This establishes entropy as a concave, albeit not strictly concave function η(ε,x), a property that is regarded as axiomatic in much of the literature on thermodynamics and continuum thermomechanics and which is reflected by the second criterion of Coleman & Noll [32] for equilibrium. This (negative) convexity is deemed essential to more general ‘entropy’ functions by Ball & Chen [57], who point out that they often represent free energies.

From the preceding results one can derive by means of Legendre–Massieu transformations other equilibrium potentials such as ψ(θ,x)/θ, which is the Legendre–Massieu dual of −η(ε,x) with respect to ε, as follows from relations given in the appendix. When various of the thermodynamic potentials suffer loss of convexity of the kind associated with phase transitions the partial derivatives can be taken to be set-valued sub-gradients associated with the resulting discontinuities [58].

We note that the ostensible convex conjugate of −ε given by the Helmholtz free energy ψ(θ,x) is not fully convex but instead represents a kind of saddle-surface, being concave with respect to θ but convex with respect to x, as shown here. The first property, representing thermal stability for fixed x, follows from the fact that

3.9 $$\partial_{\theta }\psi =-\eta \;\text{and}\;∴\;\partial_{\theta }^{2}\psi =-1/\partial_{\eta }\theta =-1/\partial_{\eta }^{2}\varepsilon <0,$$

the inequality following from the convexity of ε(η,x) with respect to η. The second property, representing configurational stability, follows from the isothermal stability condition

3.10 $$-{\left(\delta \text{f}\right)}_{\theta }\cdot \delta \text{x}=\delta \text{x}\cdot \partial_{\text{x}}^{2}\psi \cdot \delta \text{x}\geq 0$$

corresponding to the continuum-elastic convexity of Helmholtz free energy with respect to x; cf. [55,59]. It then follows that the relevant Hessian $\{\partial_{\theta },\partial_{\text{x}}\}⊗\{\partial_{\theta },\partial_{\text{x}}\}\psi$ has 1×1 and n×n diagonal-block submatrices that are, respectively, negative and positive definite, which rules out it being positive or negative definite. Most of the above conclusions are reflected in the relations given by Lubarda [55] for the special case of thermoelastic equilibria of elastic continua subject to certain boundary conditions. The convexity classification of η and ψ are readily verified for the other extreme of material behaviour, the ideal gas, for which

3.11 $$\eta =\frac{3}{2}\ln\varepsilon +\ln v+\text{const.}\;\text{and}\;\theta =\frac{2}{3}\varepsilon ,$$

where energies are scaled by the gas constant R. For the simple systems at hand the appendix provides general mathematical arguments.

With some evident loose ends, this wraps up our discussion of equilibrium and stability, of a sort that is necessary for a complete statement of the second law in its thermostatic form. We now address the non-equilibrium situation.

## 4. Non-equilibrium entropy

As is the case with the seminal work of Clausius [39], most contemporary treatments of non-equilibrium thermodynamics assume the temperature and entropy appearing in the inequality

4.1 $$\text{d}\eta \geq \frac{đq}{\theta },$$

and its continuum thermomechanical counterpart, to be primitive quantities, without regard to observability. It is, however, a truism that in many systems far from equilibrium there is no unique temperature, as e.g. is the case in non-equilibrium gas dynamics with unequal vibrational, rotational and translational temperatures, or in gaseous plasmas with different ionic and electronic temperatures, and with no known way to measure a non-conserved quantity such as entropy. (Indeed, it can be argued that most if not all physical measurements rely on a conservation law, and conserved quantities play a special role in statistical mechanics.) Thus, this author shares the long-standing concerns of Meixner [33,34] (cf. the comments of Bataille & Kestin [21] and Maugin ([22], Section 5.3) regarding *rational thermodynamics*) that most treatments of irreversible thermodynamics rest on shaky ground.

Faced with the above conceptual impasse, we concur with Meixner [46] that the only uniquely defined temperature of a non-equilibrium system is that of an equilibrium state having the same energy and configuration as the actual state, which, incidentally, is also the case with other forces or potentials borrowed from thermostatics. Hence, as a principle based on recoverable work, we propose to take the entropy η and, hence, the Helmholtz free energy ψ, in non-equilibrium states to be given by

4.2 $$\theta [\eta_{e}(\varepsilon ,\text{x})-\eta ]=\psi -\psi_{e}(\theta ,\text{x})=w_{m},\;\text{with}\;\theta =\theta_{e}(\varepsilon ,\text{x}),$$

where subscript *e* refers to the equilibrium states considered above, and where $w_{m}$ *is the theoretical maximum recoverable work that could be obtained from the system by the conversion of an equal amount of heat received from a thermal reservoir or ‘heat bath’ at constant temperature* $\theta_{e}$, *in passing from a non-equilibrium state to the equilibrium state having the same energy* ε *and configuration* x.

The recoverable work employed in the present definition of non-equilibrium entropy differs from that proposed by Lieb & Yngvason [60], which involves an environmental temperature, presumably the lowest available of the type appearing in the *exergy* employed in the practical second-law analysis of thermodynamic efficiency. Given the role of external temperatures in the classical formulations of the second law, one might argue that it makes sense to include an environmental temperature in the definition of non-equilibrium entropy. To some extent, this motivates theories based on two temperatures [46,61]. That said, the present author prefers to work with intrinsic state variables.

Assuming that $w_{m}$ is derived from observables associated solely with the state of the system, we must assume that it depends generally on the entire history of the state variables z={ε,x}, or, formally (in the spirit and notation of [62]), with t denoting time

4.3 $$w_{m}(t)=W\{\text{z}(\underset{-\infty }{\overset{t}{t^{\prime }}})\},$$

a quantity that will generally involve non-equilibrium forces $\text{f}\neq {\text{f}}_{e}$ conjugate to x and given by constitutive equations implicit in (4.4). For example, a rigid heat conductor with internal temperature gradients might require an infinite set of heat engines (e.g. thermoelectric) operating between local temperatures and $\theta_{e}$. Similarly, the relaxation to equilibrium in a spatially uniform but non-equilibrium state (as sometimes assumed to apply to non-equilibrium chemical reactions^4^) would require a similar collection of thermo- or chemo-mechanical devices possibly separate from the system itself. To avoid such macroscopic analogues of Maxwell’s dæmon, it might be expedient to introduce a dependence on finite set of ‘internal variables’ [10,21, p. 71], for example, temperature gradients, which vanish or become otherwise irrelevant in thermostatic states.

As an important exception to the situation envisaged in the preceding paragraph, one can define a class of *thermodynamically simple systems*, as an extension to non-equilibrium states of Cathéodory’s thermostatically simple systems, in which the same state variables, endowed with non-equilibrium conjugate forces, determine the work in all irreversible processes. In this case, the force conjugate to x is presumably given by a history-dependent functional f (of the type introduced in the continuum thermomechanics of Coleman [62]):

4.4 $$\text{f}(t)=f\{\text{z}(\underset{-\infty }{\overset{t}{t^{\prime }}})\},$$

with, as concrete example, the generalization of linear viscoelasticity

4.5 $$f\{\text{z}(\underset{-\infty }{\overset{t}{t^{\prime }}})\}=\int_{-\infty }^{t}m(t-t^{\prime })\dot{\text{z}}(t^{\prime })\;\text{d}t^{\prime }=\int_{0}^{\infty }m(s)\dot{\text{z}}(t-s)\;\text{d}s,$$

where m(s) denotes a (Boltzmann–Volterra) memory kernel.

In general, the maximum work that can be obtained by a constant-energy cycle beginning at the non-equilibrium state at time t and ending at an equilibrium state with the same configuration z is given by the extremum

4.6 $$\begin{array}{rl} & w_{m}(t)=\max_{{\text{x}}_{t}(s)}\int_{t}^{\infty }{\text{f}}^{+}(s)\cdot \text{d}{\text{x}}_{t}(s),\;\text{with}\;{\text{f}}^{+}(s)=f\{{\text{z}}^{+}(\underset{-\infty }{\overset{s}{t^{\prime }}})\},\\ & \text{where}\;{\text{z}}^{+}(t^{\prime })=\left\{ \begin{array}{ll} \text{z}(t^{\prime }), & \text{for}\;t^{\prime }<t,\\ \{\varepsilon (t),\text{x}(t^{\prime })\}, & \text{for}\;t^{\prime }>t \end{array}\right.\\ & \;\text{and}\;{\text{x}}_{t}(s)=\{\text{x}(s):\lim_{s\to \infty }\text{x}(s)=\text{x}(t)\},\;\text{and}\;{\text{x}}_{t}(t)=\text{x}(t). \end{array}\},$$

involving an isenergic prolongation ${\text{z}}^{+}$ of a general history. The upper limit on the integral allows formally for an asymptotic approach to equilibrium and we set aside questions of discontinuities at time t exemplified by Coleman’s [45] analysis.

The rationale behind (4.6) is virtually the same as that underlying the derivation of free energy for linear viscoelastic materials undertaken by several investigators, e.g. [35–37], although most of those analyses assume isothermal conditions. The paper by Gentili [37] offers a comprehensive review and some new results, showing that the minimum free energy is given by maximum recoverable work. At the time of this writing, the author is unaware of the connection to Coleman’s [45] free energy of impulsive elastic strains alluded to above.

It is also worth mentioning certain nonlinear viscoelastic models that involve the equivalent of time-dependent ‘potentials’ or free energies, such as the well-known K-BKZ [63] model, and variants, e.g. [64,65].^5^ While interesting, it would be beyond the scope of this paper to clarify the precise relation of such models to the optimization problem in (4.6). At any rate, the common feature of these and the present model is that the constitutive equations needed to define the history (4.4) also provide the definition of non-equilibrium entropy.

Mention should also be made of the rate-independent frictional systems and continuum plasticity discussed in the Introduction and anticipated by Gibbs [42, p. 58]. For the special case of strictly dissipative processes, such as constant friction or rigid, perfectly plastic continua, it seems appropriate to adopt the proposal of Bridgman [44] and take $\eta =\eta_{e}$ in (4.2), implying that no work can be recovered from such systems. On the other hand, elastoplasticity, like viscoelasticity, generally involves a partially elastic response to transient loading or unloading from a thermostatic or other steady state. Such forces must also be involved in Bridgman’s [44] steady-state elastoplastic hysteresis loops, which vitiates his proposal to take $\eta =\eta_{e}$, since stored elastic energy is in principle partly recoverable.

Plastic strain-hardening with back-stress, as well as related ‘shape-memory’ effects, may also involve recoverable strain energy. Since the relation (4.6) involves only configurational changes, it might require a not completely evident extension to thermal processes such as thermal annealing or phase change to recover plastically locked-in elastic energy. These considerations pose interesting problems of extremalization, which to the author’s knowledge are not addressed in the literature for anything other than the most rudimentary models of elastoplasticity. (Other approaches to the thermodynamics of plasticity involving internal variables are to found in the articles by Nemat–Nasser and Mandel in [66] and more recent publications too numerous to mention here.)

In most of the above examples dealing with extremalization of viscoelastic response, temperature rather than internal energy is held constant. The distinction may not be important for certain materials, particularly those that exhibit ‘entropic elasticity’ (e.g. rubber-like solids subject to shearing and quasi-ideal gases subject to compression).

Note that, if unrecovered, the quantity $w_{m}$ may be regarded as the ‘lost work’ (i.e. lost conversion of heat to work) identified in certain engineering analyses [67,68], whereas $-{\dot{w}}_{m}$, presumably non-negative, may provisionally be identified with dissipation rate.

The present definition of non-equilibrium entropy suggests that theories based on the history of {θ,x}, notably the continuum thermomechanics of Coleman [62], should be modified by simply replacing θ by $\theta_{e}(\varepsilon ,\text{x})$, or, more simply, by ε. Thus, instead of a hypothetical temperature θ in a *thermometric* theory, we appeal to a calorimetric measurement of ε (relative to some standard state) in an alternative *calorimetric* theory (which could also be designated as *ergometric*). Coleman’s [62] entropy functional now depends on the history of ε rather than a putative temperature θ. Within this new framework, the conventional constitutive equations for quantities such as heat and entropy flux would require modifications to the usual statement of the Clausius–Duhem inequality in order to obtain second-law restrictions, and such a programme has already been undertaken by Meixner [33,46]. Any comparable effort should lead to restrictions on constitutive equations represented by (4.4) to guarantee that recoverable work defined by (4.6) be non-negative. This is obviously connected with the condition of positive dissipation discussed, e.g. by Domingos [20], which in the current notation requires that $(\text{f }-{\text{f}}_{e})\cdot \dot{\text{x}}\leq 0$. While these are matters for further investigation, they serve to emphasize once more that non-equilibrium entropy is determined by dissipative constitutive equations, with dissipation based on a proper formulation of the Clausius–Duhem inequality.

## 5. Conclusion

Key conclusions are summarized in the abstract. To briefly elaborate here, we have presented a revision of Carathéodory’s putative derivation of the second law of thermodynamics for systems with finite state space, represented here by observables such as internal energy and a set of configuration variables that define work. This establishes entropy as integral hypersurfaces representing a foliation of the given state space, with adiabaticity equivalent to a holonomic constraint to these surfaces and absolute temperature as generalized reaction force. On the assumption of positive temperature, the inverse of the entropy function yields the caloric equation of state. Based on an alternative treatment of constraints, we also obtain a calorimetric definition of entropy based on a condition that is tantamount to the third law of thermodynamics.

Second and more important are the criteria proposed for equilibrium and stability based on work principles, as a foundation for the classical thermostatics of Gibbs and the modern versions devoted to simple continua. The first of these two principles is based on a tentative generalization of the classical condition for mechanical stability, and the second on a generalized thermomaterial work principle for stable equilibrium that is tantamount to requiring the caloric equation of state to be convex. This leads to concavity of the entropy as state function without further assumption. As an inconclusive issue subject to further investigation, there is a question as to the general validity of the first work principle and its connection to the strict convexity of the caloric equation that is demanded by Coleman and Noll.

As a final and perhaps most important contribution, we have proposed a recoverable-work principle for thermodynamic systems that provides a definition of a history-dependent non-equilibrium entropy. This *calorimetric* theory serves as alternative to the *thermometric* theory of Coleman and co-workers, where entropy is given by an unspecified functional of the history of a generally undefined temperature. The new theory establishes an appealing connection to efforts by others to derive a free energy from constitutive equations for continuum viscoelasticity. This new definition of non-equilibrium entropy requires a revision of the Clausius–Duhem inequality, a revision not undertaken in the present work.

## Footnotes

1 Max Born is generally credited with encouraging Carathéodory to undertake an axiomatic approach to the subject [2,3].

2 I cite both collections of the scientific papers of Gibbs, having recently discovered that the latter is available in digital format with searchable text.

3 This differs from Honig’s [24, p. 417] suggested derivation of the first law, which seems to be based on the argument that adiabatic work implies the existence of ε as a state function, but it is not clear what constitutes the independent state variables.

4 Chemical reactions relaxing to equilibrium should generally not require internal variables beyond the concentrations of chemical constituents that figure among the configurational variables x, endowed with conjugate forces given by the chemical potentials of Gibbs. An exception might be inaccessible chemical constituents, i.e. ‘hidden variables’, suffering disequilibrium caused by heating or working.

5 The connection of all these studies to the thermodynamics of instantaneous elastic response treated by Coleman [45] is not completely clear to this author.

## Data accessibility

This article has no additional data.

## Appendix A. Duality and convexity

Given a function f(z),Z→R on an (n+1)-dimensional space $Z=R^{n+1}$ that can be decomposed as the direct sum $Y⊕Y^{\prime }$, with $Y^{\prime }=Z\setminus Y$ denoting an m-dimensional subspace, the Legendre (or Legendre–Massieu [43, p. 336], [69]) dual with respect to y∈Y is given by

A 1 $$L_{\text{y}}\{f\}=f(\text{z})-\text{y}\cdot {\text{y}}^{*}=-f^{*}({\text{y}}^{*},{\text{y}}^{\prime }),\;\text{where}\;{\text{y}}^{*}=\partial_{\text{y}}f,\;\text{and}\;{\text{y}}^{\prime }=\text{z}\setminus \text{y},$$

where asterisks represent standard mathematical notation for convex conjugates whenever f is a convex function of y. Note that the convexity of f with respect to y is formally determined by

A 2 $${\left(\Delta L_{\text{y}}\{f\}\right)}_{{\text{y}}^{*}}=\Delta f-{\text{y}}^{*}\cdot \Delta \text{y}\geq 0.$$

The case where f is convex in y but not in ${\text{y}}^{\prime }$ represents *partial convexity* [70]. In this case $f^{*}$ is convex in ${\text{y}}^{*}$ and the Legendre dual is therefore concave in ${\text{y}}^{*}$. Note that if f(z) is convex in z, it is obviously convex in both y and ${\text{y}}^{\prime }$. It can therefore be shown that $\text{y}\cdot \partial_{\text{y}}f$ is a convex in ${\text{y}}^{\prime }$, such that $f^{*}$ is convex in ${\text{y}}^{\prime }$ and concave in ${\text{y}}^{*}=\partial_{\text{y}}f$, the situation being reversed for the Legendre dual.

Two examples relevant to the present paper, where z={η,x} and f is the convex function ε(z), are:

A 3 $$m=n+1\;\text{with}\;Y=Z,\;\text{and}\;g(\theta ,\text{f})=\varepsilon -\theta \eta +\text{f}\cdot \text{x }=-\varepsilon^{*}(\theta ,\text{f}),$$

where g, a concave function of its arguments, is the (*plenipotent*) Gibbs free energy of the type employed, e.g. in [32,55] for continuum elasticity, to be distinguished from the (*partial*) Gibbs free energies ε−θη+pv employed for pure isotropic fluids. The second example is

A 4 $$m=1,\;\text{with}\;\psi (\theta ,\text{x})=\varepsilon -\theta \eta =-\varepsilon^{*}(\theta ,\text{x}),$$

so that ψ is concave in θ and convex in x.
